# Model-based estimation of muscle and ACL forces during turning maneuvers in alpine skiing

**DOI:** 10.1038/s41598-023-35775-4

**Published:** 2023-06-03

**Authors:** Dieter Heinrich, Antonie J. van den Bogert, Martin Mössner, Werner Nachbauer

**Affiliations:** 1 Department of Sport Science, University of Innsbruck, Innsbruck, Austria; 2 Department of Mechanical Engineering, Cleveland State University, Cleveland, OH USA

**Keywords:** Motor control, Mechanical engineering

## Abstract

In alpine skiing, estimation of the muscle forces and joint loads such as the forces in the ACL of the knee are essential to quantify the loading pattern of the skier during turning maneuvers. Since direct measurement of these forces is generally not feasible, non-invasive methods based on musculoskeletal modeling should be considered. In alpine skiing, however, muscle forces and ACL forces have not been analyzed during turning maneuvers due to the lack of three dimensional musculoskeletal models. In the present study, a three dimensional musculoskeletal skier model was successfully applied to track experimental data of a professional skier. During the turning maneuver, the primary activated muscles groups of the outside leg, bearing the highest loads, were the gluteus maximus, vastus lateralis as well as the medial and lateral hamstrings. The main function of these muscles was to generate the required hip extension and knee extension moments. The gluteus maximus was also the main contributor to the hip abduction moment when the hip was highly flexed. Furthermore, the lateral hamstrings and gluteus maximus contributed to the hip external rotation moment in addition to the quadratus femoris. Peak ACL forces reached 211 N on the outside leg with the main contribution in the frontal plane due to an external knee abduction moment. Sagittal plane contributions were low due to consistently high knee flexion (> 60$^{\circ }$), substantial co-activation of the hamstrings and the ground reaction force pushing the anteriorly inclined tibia backwards with respect to the femur. In conclusion, the present musculoskeletal simulation model provides a detailed insight into the loading of a skier during turning maneuvers that might be used to analyze appropriate training loads or injury risk factors such as the speed or turn radius of the skier, changes of the equipment or neuromuscular control parameters.

## Introduction

In alpine skiing, the forces and moments acting on the skier as well as the skier’s muscle forces and joint loads are essential to assess the loading of the skier. In particular, competitive skiing is highly demanding and requires a high level of strength, endurance, cardiovascular fitness as well as technical and tactical skills^1,2^. Due to the high demands, competitive skiers face a quite high injury risk. Epidemiological data captured by the Injury Surveillance System of the Austrian Ski Federation as well as the International Ski Federation (FIS) show, that on average about every seventh to ninth athlete suffers a severe injury every season^3–5^. The most common injured body part is the knee accounting for about two-thirds of all severe injuries^3,5^. At the knee, severe injuries typically involve the knee ligaments and a torn anterior cruciate ligament (ACL) is the most frequent diagnosis accounted for 70.8% of severe knee injury events and 48.6% of the overall severe injury events^5^. Injured athletes suffer from a number of short-term and long-term consequences such as knee instabilities or an increased risk of osteoarthritis^6^ and ACL re-injury^7^. As a consequence, the prevention of severe knee injuries and the development of effective injury prevention programs is of utmost importance.

To develop effective injury prevention programs a detailed understanding of the events leading to injury and the injury mechanisms is required^8^. Typical situations leading to severe injuries in alpine skiing are jump landing and turning maneuvers^9,10^. Video analysis is a common tool to analyze injury-prone situations^11^ and has advanced our understanding concerning the kinematics of the athlete during injury cases such as during jump landing and turning maneuvers in alpine skiing^9^. However, video analysis does not provide information about the underlying muscles forces, the exact time of injury, or the ligament forces. In particular, muscles have the ability to increase or decrease the mechanical loads on the ACL, and thus are viable targets for preventative interventions^12^. Unfortunately, direct measurement of muscle forces is generally not feasible and non-invasive methods based on musculoskeletal modeling should therefore be considered^13^.

Musculoskeletal modeling is a viable tool to estimate muscle forces and soft tissue loading non-invasively^13^. In previous research, several studies have applied musculoskeletal models successfully to investigate muscle forces and ACL forces during dynamics activities such as jump landing, sidestep cutting or stop-jump maneuvers^14–16^. In alpine skiing, however, musculoskeletal modeling has only been applied to analyze muscle and ligament forces during jump landing maneuvers and selected risk factors such as backward lean^17,18^, the landing height of the skier^19^ or the stiffness of the ski boots^20^. Furthermore, all of these studies used a two dimensional musculoskeletal skier model and focused on the sagittal plane of the landing maneuver. Simulation of a turning maneuver, however, requires a three dimensional model and to the authors’ knowledge, muscle and knee ligament forces (i.e. ACL forces) during a turning maneuver have not been investigated so far.

Therefore, the first objective of the present study was to estimate the muscle and knee ligament forces during turning in alpine skiing using a three dimensional musculoskeletal skier model. Regarding the knee ligament forces, we focused on the force in the ACL, since ACL injuries constitute the most common type of severe injuries in alpine skiing. The second objective was to establish a realistic simulation as a starting point for further studies investigating out of balance and injury prone situations as well as corresponding risk factors.

## Methods

### Musculoskeletal skier model

To investigate the muscle forces during a turning maneuver, we used a recently developed three dimensional musculoskeletal model of an alpine skier^21^. The skeletal model of the skier was based on the full-body OpenSim model of Hamner et al.^22^ and had 19 degrees of freedom (6 between pelvis and ground; 3, 1, and 1 at each hip, knee and ankle joint, respectively; 3 at the lumbar joint between trunk and pelvis). The subtalar and mtp joints were locked, because a ski boot allows only a limited dorsiflexion/plantarflexion motion at the ankle joint. Stiffness properties of the ski boot were considered, incorporating a nonlinear passive moment acting at the ankle joint^20^. To decrease computational time, the position of the arms of the skier was constrained in a typical position and the mass of the ski poles was neglected.

The model of the skier included 94 three-element Hill-type muscles (43 per leg and 8 for the lumbar joint) actuating the hip, knee, ankle and lumbar joints of the skier. The chosen set of muscles was based on the OpenSim model of Catelli et al.^23^, where we added eight muscles (2 x erector spinae, rectus abdominus, external obliques, internal obliques) to actuate the lumbar joint of the skier and three hip muscles (gemelli, pectineus, quadratus femoris) to each leg to increase hip muscle strength. Muscle contraction dynamics and activation dynamics were modeled as in Nitschke et al.^24^.

Each of the two skis was split into 18 rigid segments, which were connected by revolute joints and spring-damper elements. The parameters of the spring-damper elements as well as the mass of the ski segments and related geometric properties (camber, height and width) were derived from laboratory measurements^25^. Torsion of the ski segments was neglected, because it was observed to be low during turning maneuvers^25,26^. The contact between the ski segments and the snow was modeled using three force components acting on every ski segment and taking into account the side cut shape of the ski^27,28^. The three force components were a penetration force acting normal to the snow surface, a shear force acting parallel to the snow surface and orthogonal to the ski edge and finally a friction force. Specifically, the penetration force was modeled as a function of the penetration depth and speed of the ski edge into the snow surface and the edging angle and incorporated a hypoplastic constitutive Eq.^27^. The hypoplastic constitutive equation takes into account that if the penetration depth increases along the ski and the snow is increasingly compressed, the snow remains compressed. Consequently, the hypoplastic constitutive equation allows to model the behaviour that during a carved turning maneuver in skiing the front part of the ski typically forms a snow groove and the rear part of the ski follows the groove^27^. Further details of the hypoplastic constitutive equation are provided in the supplementary information S1. The shear force provided resistance against lateral skidding of the skier proportional to the penetration depth of the ski edge into the snow surface and acted orthogonal to the ski edge. The frictional force acted antiparallel to the ski segment’s velocity with a constant friction coefficient $\mu = 0.08$. Finally, we applied air drag on the skier acting on the center of mass of the pelvis segment as this segment was the closest to the model’s centre of mass^29^. Specifically, we assumed a mean drag area $C_dA$ m^2^ of 0.3^30^, air density $\rho$ of 1.07 kg/m^3^ (dry air at 1564 m and 0$^{\circ }$C) and no wind speed.

The dynamics of the musculoskeletal skier model and the skis was given by the multibody dynamics of the skier and ski segments, the muscle contraction dynamics and the muscle activation dynamics. The controls *u* of the musculoskeletal skier model were represented by the neural muscle excitations of the muscles. The states $x=[q,\dot{q},s,a]^T$ of the musculoskeletal skier model were represented by the generalized coordinates *q* and generalized velocities $\dot{q}$ of the multibody model of the skier and skis, the projected length of the muscle fibers *s* (=length of the contractile element in the Hill muscle model) and the muscle activations *a*^31^. The system dynamics was formulated in implicit form, which was shown to result in better convergence and decreased computational time^24,31,32^. In compact form the system dynamics can be written in the following form:

1 $$\begin{aligned} f(x,\dot{x},u) = 0 \end{aligned}$$

A detailed description of the system dynamics is presented in van den Bogert et al.^31^.

### Experimental data

One male professional skier (age: 29 years, height: 190 cm, mass: 72 kg), who had competed at the FIS level, participated in the present study. The study was approved by the Institutional Review Board of the University of Innsbruck and all methods were performed in accordance with the relevant guidelines and regulations. The participant gave informed consent before participating.

**Figure 1:**
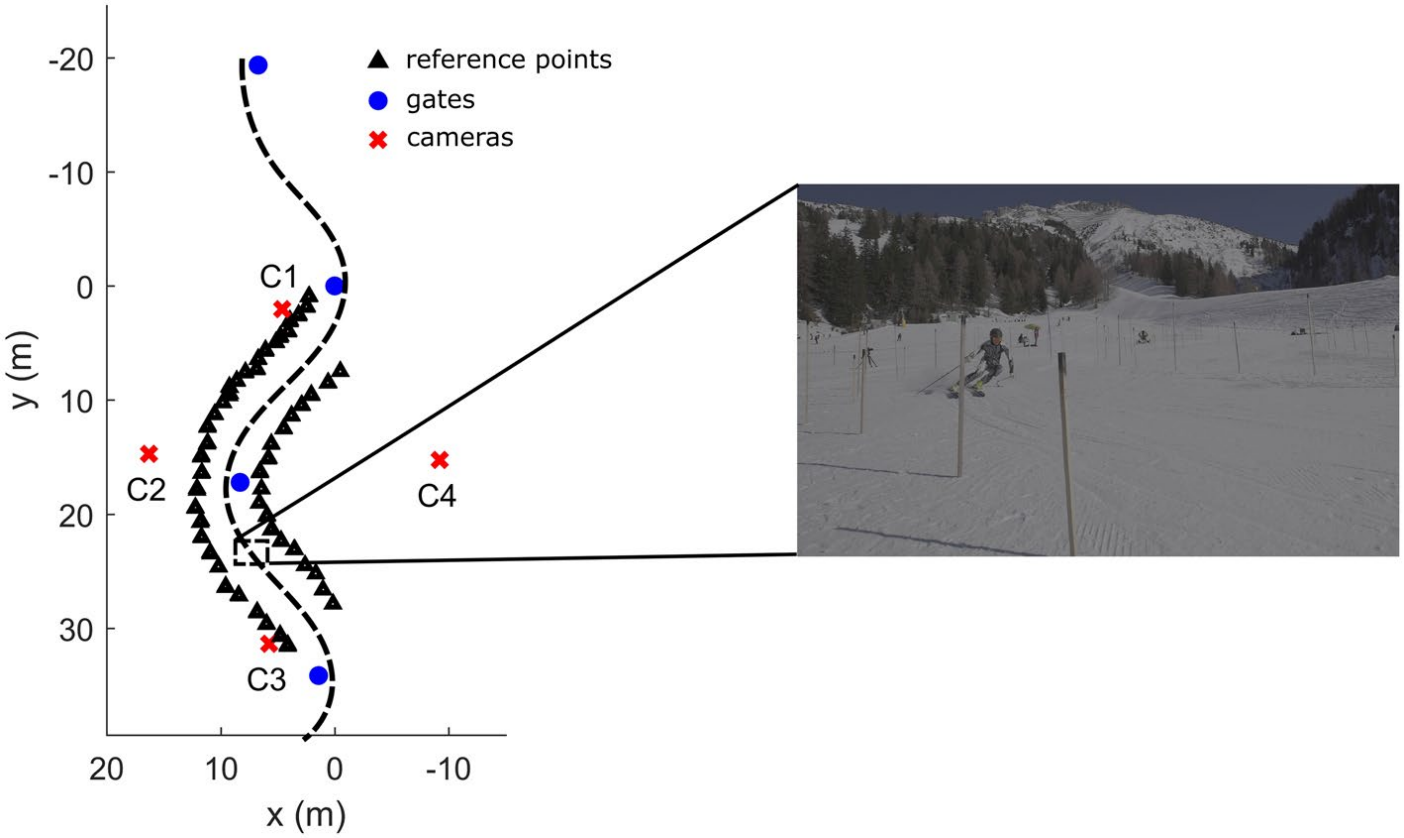
Experimental setup consisting of a sequence of 4 turns and correspondingly four gates. During the third turn the skier was captured by four cameras (C1–C4). The sample image on the right is taken from camera C3.

The experimental data were captured at the ski resort Axamer Lizum. First, the skier performed a 25 min warm up program consisting of long and short turns of his choice. After that, the skier performed three trials on a given course, which was used for data acquisition (Fig. 1). Specifically, starting with a straight schussing maneuver, the skier performed a sequence of 4 turns (displacement of the gates: 8 m in transverse direction and 16 m in downhill direction, slope angle 12.3$^{\circ }$) using carving skis with a side cut radius of 18 m. The straight schussing section and the first two turns were used to take up speed and to get used to the rhythm of the turn sequence. During the third turn the motion of the skier was captured by a multi-camera system consisting of four synchronized Sony Alpha A7s III video cameras with a sampling frequency of 100 Hz. The capture volume of the third turn was surrounded by 56 wooden poles, which were geodetically surveyed at the top and bottom by a theodolite (with an accuracy of 3–5 mm) to obtain the three dimensional coordinates of 112 reference points in a global coordinate system. The 56 reference points at the bottom were used to generate a function $z=s(x,y)$ approximating the snow surface. After data collection, 31 keypoints of the skier as well as surrounding reference points were manually digitized for each camera and each frame. Given the digitized reference points and the corresponding measured three dimensional coordinates, the three dimensional coordinates of the skier’s keypoints were computed using the direct linear transformation (DLT) method. The accuracy of the DLT reconstruction was assessed by comparing the positions of reconstructed reference points not used in the calibration of the cameras and the positions obtained by geodetic surveying. Mean errors were below 2 cm when at least 8 reference points were used for the calibration^33^. The three dimensional (3D) keypoints were approximated with smoothing quintic splines.

Given the 3D keypoints of the skier, we used the scale tool of OpenSim^34^ to scale the musculoskeletal skier model and used the inverse kinematics tool to compute the kinematics of the skier (i.e., joint angles of the inside and outside leg, joint angles at the lumbar joint and the global translation and orientation of the pelvis segment of the skier).

Parallel to the motion of the skier, EMG data of ten muscles of the outside right leg were collected using a wireless Noraxon Ultium EMG system (Noraxon USA Inc., Scottsdale, AZ, USA) with a sampling rate of 2000 Hz and a bandpass filter (10–500 Hz). Surface electrodes were placed on the tibialis anterior, gastrocnemius medialis, vastus medialis, rectus femoris, vastus lateralis, biceps femoris (long head), semitendinosus, adductor longus, gluteus medius and gluteus maximus. Before placing the surface electrodes, the skin was carefully shaved, abraded and cleaned with alcohol. EMG electrodes were placed on the muscle belly of each muscle and in line with the muscle’s fiber orientation following the recommendations of Perotto et al.^35^. EMG data were full-wave rectified and low-pass filtered using a zero-lag, second-order dual-pass butterworth filter with a cut-off frequency of 6 Hz.

### Simulation of turning maneuver

To estimate the muscle and ACL forces during the captured turning maneuver, we formulated an optimization problem with the primary task to track the experimental data (i.e., pelvic translation and rotation, joint angles at the lumbar, hip, knee and ankle joints) as good as possible using the musculoskeletal skier model. In particular, we formulated a corresponding optimal control problem with a given objective function *J* in the following form^21^:

2 $$\begin{aligned} J = \frac{1}{T}\int _0^T \underbrace{\frac{w_1}{n_d} ||err_d||_2^2}_{tracking\ error} + \underbrace{\frac{w_2}{n_m} ||a||_2^2}_{muscle\ effort} + \underbrace{\frac{w_3}{n_{xu}} (||\dot{x}||_2^2 + ||\dot{u}||_2^2)}_{regularization} dt \end{aligned}$$

The objective function *J* consisted of three parts.The first part denoted the squared error between the experimental and simulated data (tracking error), where $||-||_2$ represents the Euclidean norm and $err_d$ the difference between the experimental and simulated data (i.e., joint angles of the inside and outside leg, joint angles at the lumbar joint and the global translation and orientation of the pelvis segment of the skier). Before computing the difference between the experimental and simulated data, the data were normalized such that all components were approximately of the same order. The second part (muscle effort) was used to resolve muscle redundancy. Specifically, we used the sum of squared muscles activations as a surrogate for muscle effort as in number of other studies mimicking dynamic movement tasks such as squatting^23^, jump landing^14^ or cutting maneuvers^36^. The third part was used to improve convergence of the optimization using a regularization term including the derivative of the states $\dot{x}$ and controls $\dot{u}$ of the musculoskeletal skier model^24^. Simulation time is denoted by *T* and $w_1$, $w_2$ and $w_3$ represent weight factors. The constants $n_d$, $n_m$ and $n_{xu}$ denote the number of generalized coordinates to be tracked, the number of muscles and the sum of the number of state and control variables of the musculoskeletal skier model, respectively. The optimal control problem was subjected to several constraints, namely the system dynamics of the musculoskeletal skier model (equation 1) as well as upper and lower bounds on the states *x* and controls *u* of the skier model^21,31^.

To solve the optimal control problem we transformed it into a constrained nonlinear programming problem (NLP) using the method of direct collocation, 75 mesh points and the implicit Euler formula^31^. As initial guess we used a schussing simulation of the skier in an upright position. To decrease computational time, we computed analytical derivatives of the objective function and constraints using the symbolic software package MotionGenesis (Motion Genesis LLC, Menlo Park, CA, USA) and provided these to the NLP solver IPOPT^37^.

### Data analyses

To evaluate the simulation of the turning maneuvers, we first calculated the root mean squared difference (RMSD) between each joint angle of the skier derived from the measurement data and the corresponding joint angle of the skier in the simulation. Second, we compared the speed and the track of the skier where we used the ankle joint centers of the outside and inside leg as reference points for the track^38^. Then we computed the joint moments, muscle forces and knee ligament forces of the skier. Computed muscle activation patterns were compared to the experimental EMG data. Specifically, we scaled the EMG data to the maximum of the estimated muscle activation in accordance with other studies^15,16,24^. Joint moments were represented as internal joint moments and hip flexion, adduction and internal rotation, knee extension and ankle dorsiflexion moments were denoted as positive.

Knee ligament forces were estimated using a data-driven knee model^36,39^. In the data-driven knee model, the ACL force was computed as the sum of three components corresponding to the sagittal plane, frontal plane and transverse plane, respectively. Specifically, the ACL loading component in the sagittal plane was estimated as a proportion of the anteroposterior ligamentous shear force based on cadaver knee measurements^39^. Estimates of the frontal plane and transverse plane ACL loading components were also based on cadaver knee measurements and were expressed as exponential functions of the respective joint moments (in the frontal and transverse plane) as well as the knee flexion angle^39^. Input to the knee model were the intersegmental knee joint moments and forces, the knee muscle forces and their line of action as well as the knee flexion angle during the turning maneuver. Given these input data, the ACL loading components in the sagittal, frontal and transverse planes and the total ACL force were calculated. The intersegmental joint moments and forces at the knee were computed, solving the Newton-Euler equations consecutively starting at the ski segments to the shank segment of the outside and inside leg, respectively^40^. The lines of action of the knee muscle forces were derived from the OpenSim model of Cantelli et al.^23^.

### Sensitivity study

We conducted a sensitivity study to further evaluate the sensitivity of our turning simulation to different parameters^41,42^. First, we evaluated how the muscle effort term in the objective function affects the estimated muscle and ACL forces. Although muscle activation squared (i.e. $a^2$) is a common criterion to resolve muscle redundancy during dynamic movements tasks, higher exponents have also been suggested in the literature^13,43–46^. A higher exponent penalizes peak muscle activations more strongly and is expected to result in a more fatigue-like criterion^43^. So, we changed $a^2$, to $a^3$ and $a^5$ in the muscle effort term of the objective function. Second, we evaluated how the weighting coefficient $w_2$ of the muscle effort term affects the estimated muscle and ACL forces. We altered the nominal value of 10 by a factor of 4 increasing it to 20 and 40 as well as decreasing it to 5 and 2.5, respectively. Decreasing the weighting factor is expected to result in higher muscle forces and a lower tracking error, while increasing the weighting factor is expected to result in lower muscle forces and a higher tracking error. Third, we tested the effect of the choice of the initial guess of the optimization on the simulation results. In the nominal simulation, we used a schussing simulation as initial guess, that is independent of the experimental data. As an alternative, we evaluated an initial guess that is derived from the experimental data. Specifically, we used a PD controller to track the experimental joint angles of the skier followed by a muscle redundancy solver similar to de Groote et al.^32^ to obtain the states *x* and controls *u* of the alternative initial guess. Fourth, we evaluated the influence of the number of mesh points using 50, 100 and 125 instead of 75 in the optimization.

## Results

**Figure 2:**
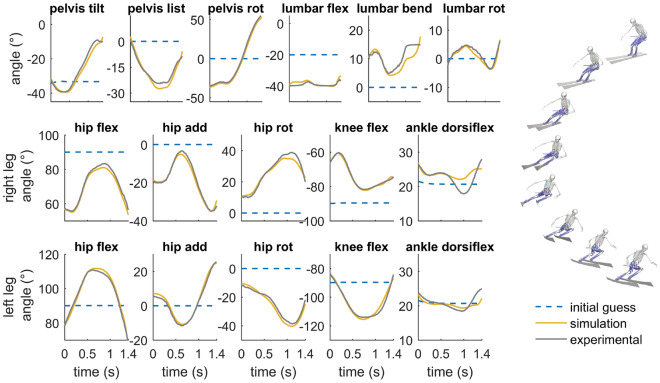
Optimized kinematics of the skier (yellow) during the turning simulation and the corresponding measured kinematic data (gray). Kinematic data refer to joint angles of the right outside leg and left inside leg, respectively, at the hip joint (flexion, adduction, internal rotation), knee joint (extension) and ankle joint (dorsiflexion). Additionally, the orientation of the pelvis (tilt, list, rotation) and the joint angles at the lumbar joint (extension, lateral bending, rotation) are shown. The blue dashed lines represent the kinematics of a straight schussing maneuver, which was used as initial guess in the optimization.

The simulation of the turning maneuver was solved in about 40 min on a single core of a workstation (Thinkstation 330, 3.5 Ghz, E-2146 CPU). The speed of the skier was about 12 m/s and increased slightly during the turning maneuvers. The minimum turn radius was about 10.7 m. The simulated turning maneuver closely resembled the experimental data with a RMSD below 2.5$^{\circ }$ for all joint angles (Fig. 2). The track of the skier also agreed very well with the measurement data with a RMSD of 0.021 m and 0.026 m at the outside and inside leg, respectively (see Supplementary Fig. S1 online).

**Figure 3:**
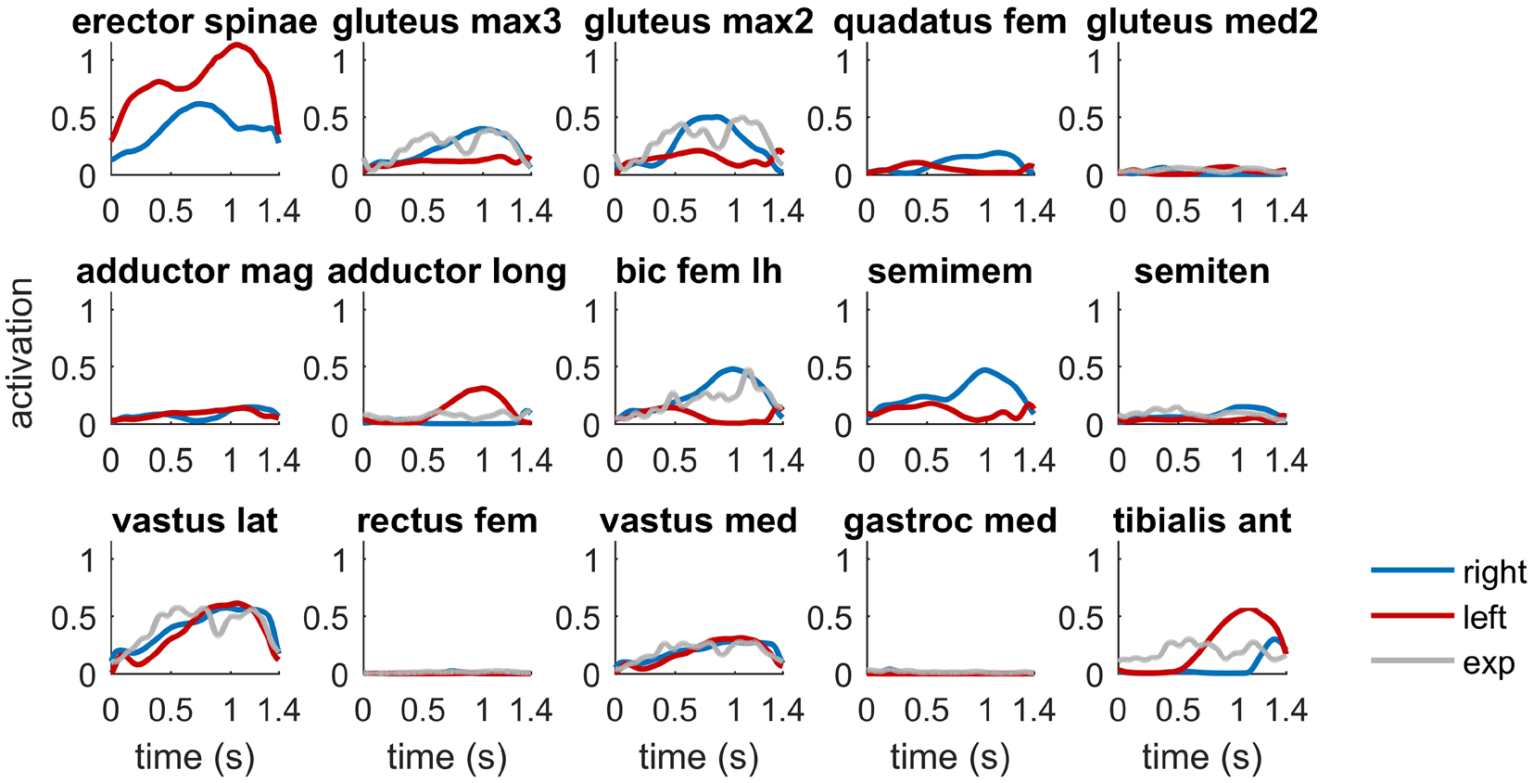
Muscle activation pattern of the primary activated muscles at the right outside leg (blue) and left inside leg (red), respectively. The measured EMG signals (exp, on the right outside leg) were scaled to the maximum activation for every muscle.

During the turning maneuver the primary activated trunk muscles were the erector spinae as well as the internal obliques. At the outside right leg, we observed the highest activations at the vastus lateralis followed by the gluteus maximus and the medial and lateral hamstrings. At the inside left leg, we observed the highest activations also at the vastus lateralis, however followed by the tibialis anterior, the adductor longus and the vastus medialis (Fig. 3). At the outside and inside leg, peak activations reached up to 61%; at the trunk the erector spinae reached the maximum activation of 100%. When compared to the experimental EMG data captured at the right outside leg, the corresponding estimated muscle activation patterns were similar during the turning maneuver for the primary activated muscle groups. Especially, the activation patterns of the gluteus maximus, biceps femoris (long head), vastus medialis and lateralis showed good agreement (Fig. 3). The complete set of muscle activations is provided in the supplementary information (see Supplementary Fig. S2 online).

**Figure 4:**
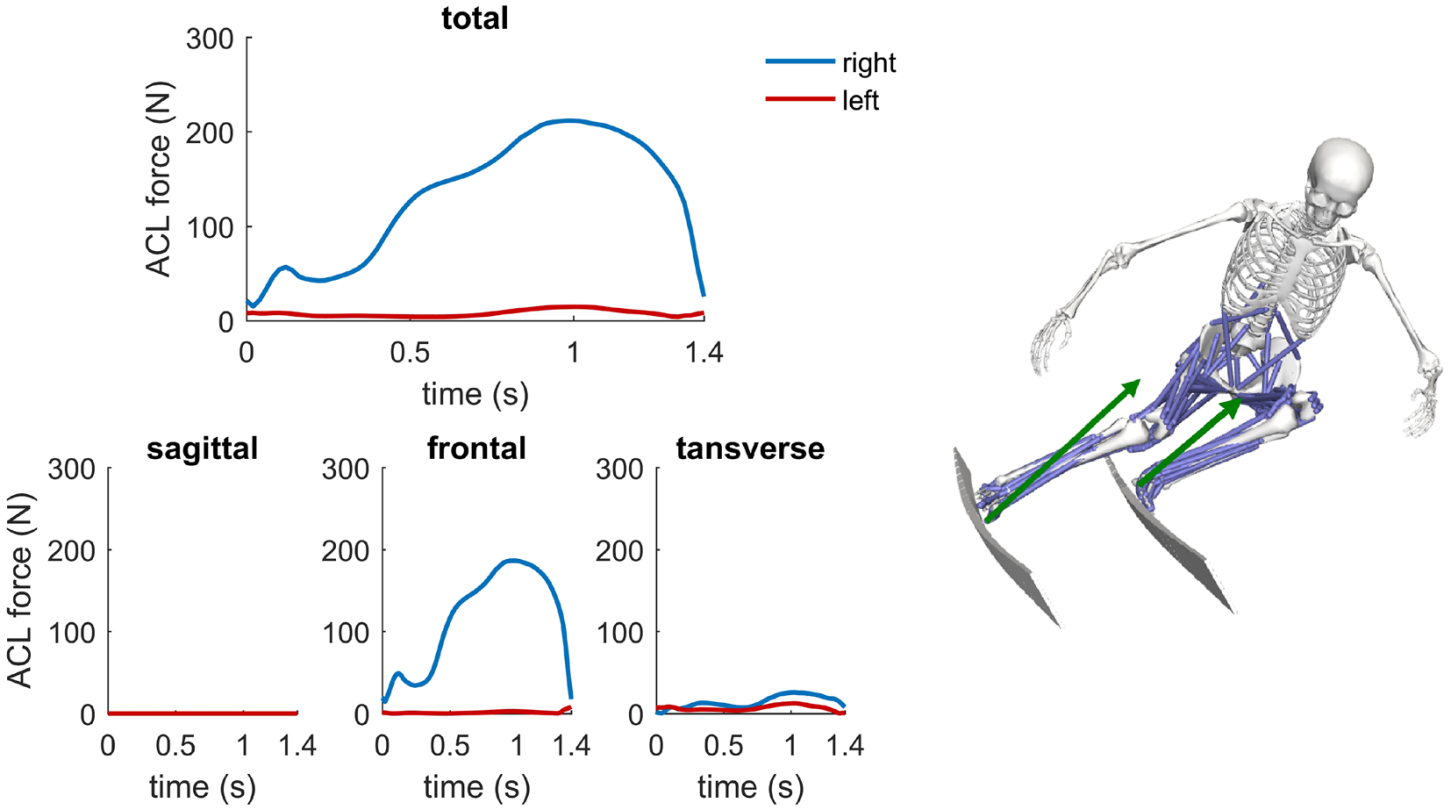
Total ACL force at the right knee (blue) and left knee (red) computed as the sum of the contributions from the sagittal, frontal and transverse planes. On the right, the green arrows represents the total ground reaction force acting on the outside ski and inside ski, respectively.

The ACL force increased throughout the turn and peaked at about 1.0 s during the steering phase of the turning maneuver. Peak ACL force was quite low amounting to 211 N on the outside leg and to 15 N on the inside leg. Analyzing the contributions from the sagittal, frontal and transverse planes, the main contribution came from the frontal plane (Fig. 4), mainly induced by the ground reaction force passing laterally to the right knee. The contributions from the sagittal plane due to an anterior shear force and from the transverse plane were very low.

**Figure 5:**
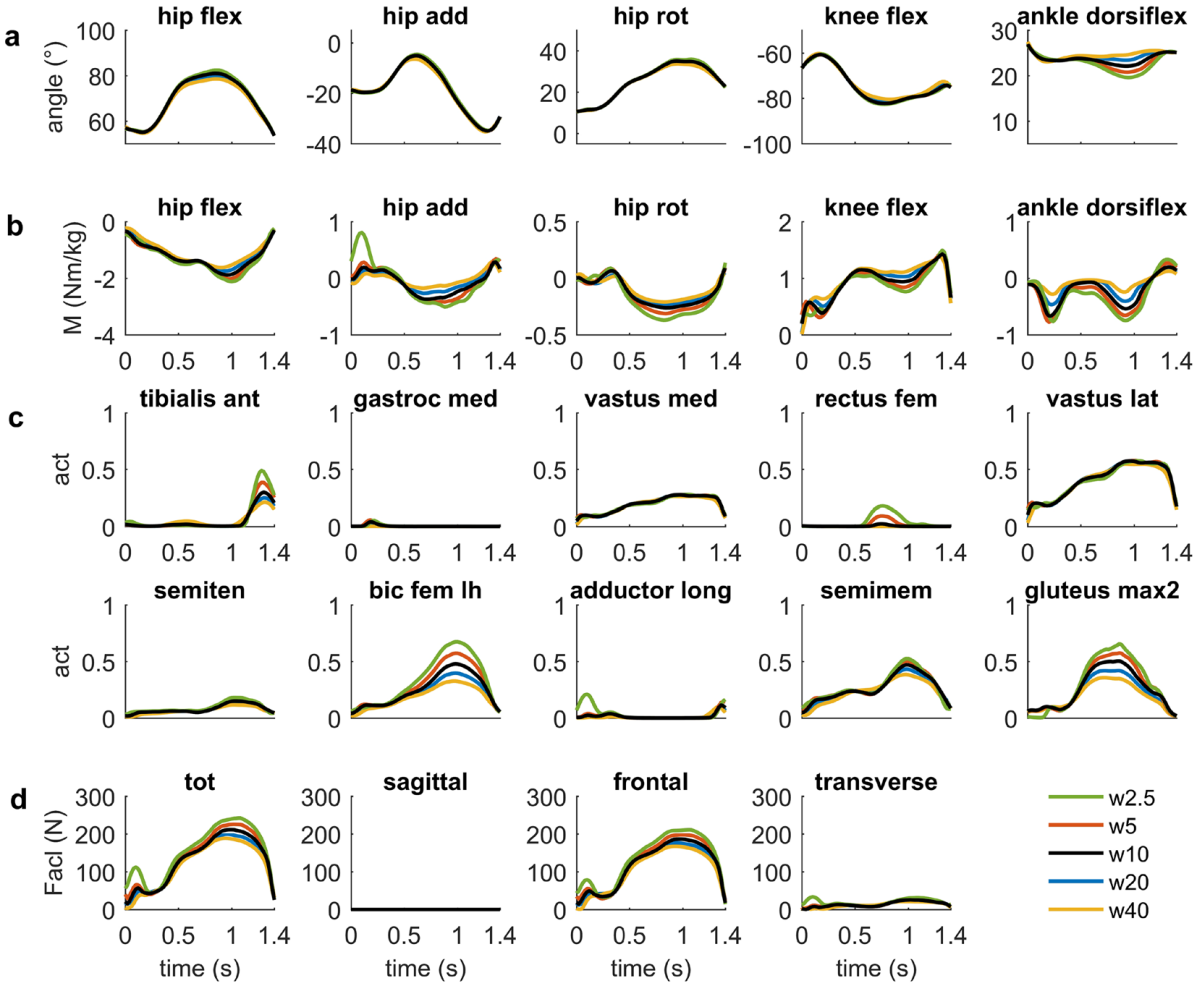
Sensitivity study analyzing the effect of the weighting coefficient (i.e., $w_2$) of the muscle effort term on the joint angles (**a**), joint moments (**b**), muscle activation patterns (**c**) and ACL forces (**d**) of the right outer leg. The weighting coefficient was varied in the range from 2.5 to 40 (w2.5–w40). Results refer only to the right outside leg, where the peak ACL force was observed.

**Figure 6:**
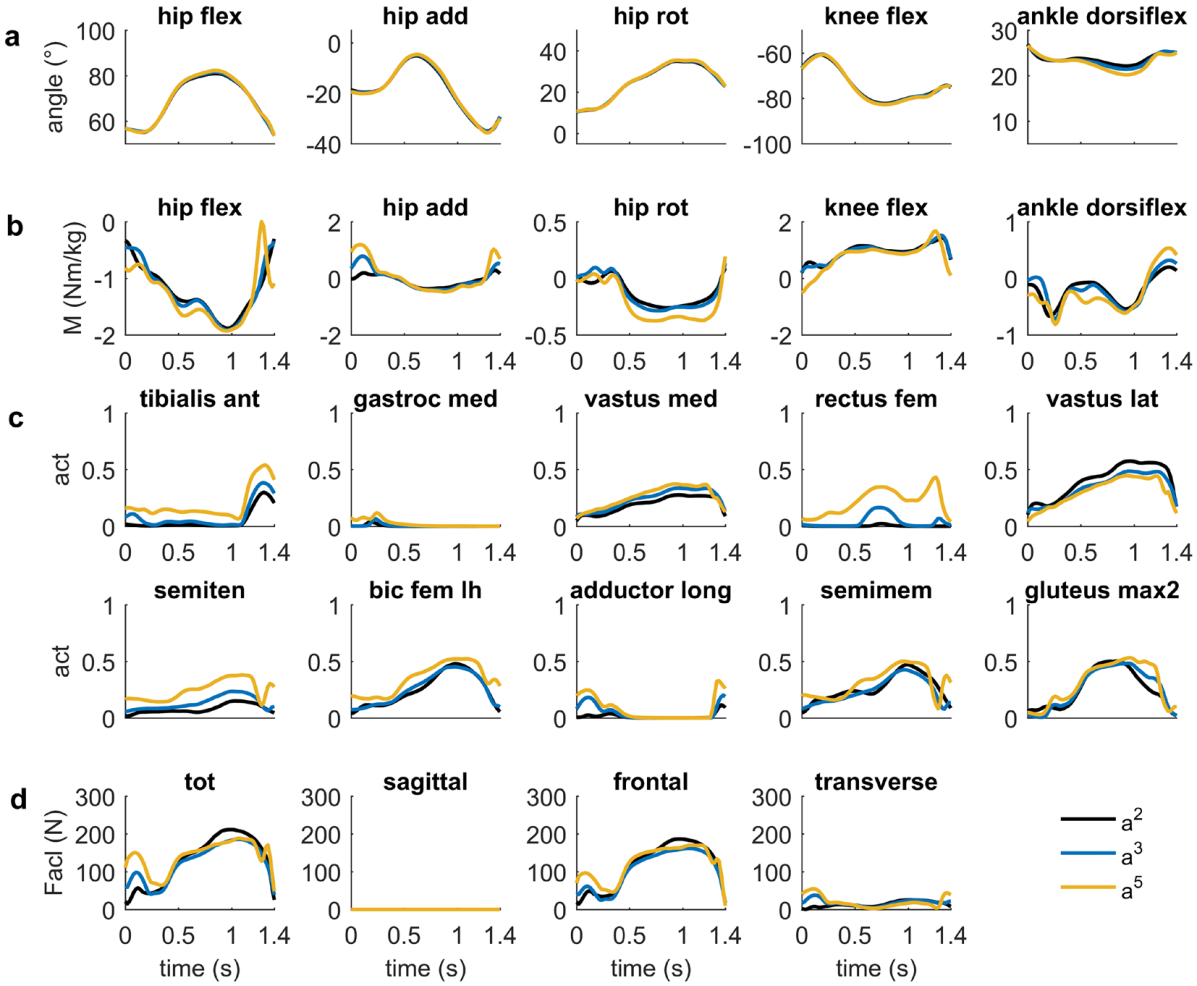
Sensitivity study analyzing the effect of the choice of the muscle effort term in the optimization on the joint angles (**a**), joint moments (**b**), muscle activation patterns (**c**) and ACL forces (**d**) of the right outer leg. Specifically, muscle redundancy was resolved minimizing the sum of squared muscle activation patterns ($a^2$), cubed muscle activations ($a^3$) and muscle activation to the power of 5 ($a^5$). Results are shown only for the right outside leg, where the peak ACL force was observed.

In the sensitivity study, decreasing the weighting of the muscle effort term (=$w_2$) in the objective function resulted in higher muscle activations during the turning maneuvers and a slightly closer fit with respect to the experimental data (Fig. 5). At the right leg, in particular, selected muscles spanning the hip joint (e.g., biceps femoris and gluteus maximus) and ankle joint (e.g., tibialis anterior) were higher activated. ACL force increased in parallel with a maximum value of 244 N, when the weighting was reduced to $w_2 = 2.5$. Altering the exponent of the muscle effort term, affected the distribution of the activated muscles. Specifically, at the knee joint of the outside leg the rectus femoris and the semitendinosus muscles were higher activated using a higher exponent (Fig. 6). Peak ACL, however, changed less markedly and reached 170 N during the turning maneuvers increasing the exponent of *a* from 2 to 5. Increasing or decreasing the number of mesh points had only a marginal effect on the kinematics of the skier, the underlying muscle forces and the ACL force (see Supplementary Fig. S4 online). An even lower effect was observed for the choice of initial guess that was used in the optimization. Here the results were almost identical (see Supplementary Fig. S5 online).

## Discussion

The purpose of the present study was to estimate the muscle and ACL forces during a turning maneuver in alpine skiing. Since, direct measurement of muscle forces is generally not feasible, we applied a musculoskeletal model of an alpine skier to track experiment data of a skier performing a turning maneuver and computed the underlying muscle forces. Subsequently, we applied a data-driven knee model to compute the force in the ACL of the knee.

The experimental data were successfully tracked by the musculoskeletal skier model in about 40 min of computational time. Solving a tracking problem is computational challenging^24,29,42,47^. In the last few years collocation methods have gained increasing popularity and have been applied in a number of studies to estimate muscle forces during dynamic movements such as walking, running, sprinting, pedaling or jump landing^19,24,29,42,47^. To the best of the authors’ knowledge, this study is the first to estimate muscle and ACL forces during a turning maneuver in alpine skiing. A turning maneuver in alpine skiing faces several challenges such as the stability of the skier, modeling the alpine skis and ski boots and the ski-snow contact. The present musculoskeletal model was similar to the recently developed three dimensional model of Heinrich et al.^21^. Compared to Heinrich et al.^21^, we modified the model of the ski-snow contact regarding the simulation of a carved turn with low lateral shearing. Specifically, we took into account that during a carved turn the front part of the ski typically forms a snow groove and the rear part of the ski follows the groove modelling the penetration force by a hypoplastic constitutive equation^27^.

The simulated turning maneuver tracked the experimental data with a RMSD below 2.5$^{\circ }$ for all joint angles and a RMSD below 3 cm with respect to the track of the skier. A similar RMSD has been obtained in our previous study^21^ estimating joint moments during a turning maneuvers and is considered to be low, because it is well within the precision of current mobile measurement devices such as machine learning techniques^48^ or inertial measurement units (IMU) based systems^49^. In addition, a sensitivity study revealed that the simulation results (i.e., kinematics, muscle forces and ACL forces) were robust with respect to the choice of the initial guess as well as the number of mesh points used in the dynamic optimization. Because we observed slight oscillations in the skier’s kinematics at 50 mesh points, that disappeared as the number of mesh points increased, 75 mesh points was the preferred choice.

During the tracking simulation of the turning maneuver, the highest activated muscles of the outside leg were the gluteus maximus, vastus lateralis as well as the medial and lateral hamstrings. The main function of these muscles was to generate the required hip and knee extension moments during the turning maneuver. In addition, the gluteus maximus was the main contributor to the internal abduction moment at the deeply flexed hip (see Supplementary Fig. S3 online). Furthermore, the lateral hamstrings and gluteus maximus contributed to the hip external rotation moment in addition to the quadratus femoris (see Supplementary Fig. S3 online). At the inside left leg we observed the highest activations also at the vastus lateralis (Fig. 3). Although the ground reaction force was lower on the inside leg, the required activation was comparable to the outside leg due to the higher knee flexion angle. The tibialis anterior was increasingly activated after the skier was passing the fall line indicating that the skier was pushing against the shaft of the ski boot. The adductor longus was the main contributor to the internal adduction moment acting at the hip joint of the inside leg (see Supplementary Fig. S3 online).

At the right outside leg, the estimated activation patterns of the primary activated muscle groups were similar to the experimental EMG data. In particular the gluteus maximus, biceps femoris (long head) and the vasti (medialis and lateralis) were in good agreement with the experimental data (Fig. 3). Compared to the vastus lateralis and medialis, the rectus femoris was hardly activated in the simulation. In the sensitivity study the activation of the rectus femoris was affected altering the muscle effort term in the objection function of the optimization (Fig. 6). Specifically, the rectus femoris was higher activated using a more fatigue-like criterion (i.e., increasing the exponent of the muscle activation *a* from 2 to 5 in the muscle effort term). Using a fatigue-like criterion, high muscle activations were more penalized and a more balanced distribution among synergistic muscles was favoured^43^. Previous EMG studies support that the biarticular rectus femoris is lower activated during a turning maneuver due to the high hip flexion encountered during skiing^50,51^. The high hip flexion puts the rectus femoris in a less favourable position because the muscle fibers are shortened below their optimal muscle fiber length^51^. However, using the sum of squared muscle activations to resolve muscle redundancy as in a number of other simulation studies^14,23,36^ likely underestimated the activation of the rectus femoris during the simulated turning maneuver. Consequently, a fatigue-like criterion might be preferable. Further research involving a larger sample size, however, is necessary to support this suggestion.

During the turning maneuver, ACL forces increased throughout the steering phase of the turn with a peak value of 211 N (= 0.3 BW) on the right outside leg. The sensitivity study showed that peak ACL force was quite robust and did not exceed 244 N in all simulations. The main contribution to the ACL force on the outside leg was in the frontal plane due to an external abduction moment. The external abduction moment was mainly caused by the ground reaction force, which passed laterally to the knee (see Fig. 4). Interestingly, the contribution to the total ACL force from the sagittal plane due to an anterior shear force was very low. This can be explained by the ground reaction force, the knee flexion angle as well as the knee muscles forces. Specifically, the ground reaction force acted posterior onto the tibia due the ski-snow friction pushing the anteriorly inclined tibia backwards. In addition, the knee flexion was always higher than 60 $^{\circ }$ and the medial and lateral hamstrings were substantially co-activated inducing a posteriorly directed shear force acting onto the tibia^12^. On the inside leg a remarkably lower ACL force occurred during the turning maneuver. Main differences between the outside and inside leg were, that the knee was higher flexed and that the ground reaction force was (a) on average 36% lower on the inside leg and (b) passed medially and posteriorly to knee inducing an external knee adduction moment (Fig. 4).

Compared to peak ACL forces estimated during jump landing maneuvers in competitive downhill skiing, the present values are substantially lower^18,19^. In particular, peak ACL values of 0.9 BW^18^ and 1.27 BW^19^ were reported, respectively, for landing heights of 0.9 m and 1.29 m and knee flexion angles of 35$^{\circ }$ and 33$^{\circ }$ at the time of peak ACL force. The differences might be explained by the decreased knee flexion angles at the time of peak ACL force and the higher tibiofemoral compression forces during jump landing, which elevate the anterior tibial shear force and consequently the peak ACL force due to the tibial plateau angle^18^. Interestingly, in the frontal plane similar peak ACL forces have been reported during sidestep cutting maneuvers, which are common dynamic movement tasks in other sports. Specifically, Weinhandl et al.^52^ reported peak ACL values of 0.3 BW in the frontal plane during anticipated and unanticipated sidestep cutting tasks. In the sagittal plane, however, peak ACL forces were substantially larger as in the present study amounting to 0.7 BW during anticipated sidestep cutting and to 0.8 BW during unanticipated sidestep cutting. Compared to the present study the hip flexion, knee flexion and ankle dorsiflexion values were considerably lower during sidestep cutting at the time of peak ACL force, which might have contributed to the increased peak ACL force in the sagittal plane.

Some limitations of the present study should be noted. First, in the simulation of the turning maneuver, we did not track the movement of the arms. The main reason was to decrease the complexity of the skier model and consequently computational time, which is a main burden of dynamic optimizations involving a three dimensional musculoskeletal model^24,41,47^. We expect, however, that the impact on the computed muscle and ACL forces is low, since we included the mass and inertia properties of the arms in the model and assumed a mean posture of the arms during the turning simulation.

Second, in the present study, we analyzed muscle and ACL forces of a skier, performing a well balanced regular turning maneuver and modeled the knee with a one degree of freedom model. Using a one degree of freedom model, we would like to note, that there was still a three dimensional intersegmental force and moment acting at the knee joint. The reason for modelling the knee joint with one degree of freedom was that flexion/extension is the only rotational degree of freedom that is mainly controlled actively by muscles. For the other two rotational degrees of freedom, passive mechanisms are much more important. Consequently, a standard approach is to assume that they are controlled passively^53^ and to use a knee model with one degree of freedom. Knee models with one degree of freedom have also been used in a number of other simulation studies involving dynamic tasks such as stop-jump^16^ or cutting maneuvers^15^.

Third, in the present study we investigated a turning maneuver of professional skier performing a carved turn with a minimum turning radius of about 10.7 m. Altering the kinematic input data, the ski properties and subject-specific parameters of the skier, the present computational framework might be applied to study turning maneuvers in selected skiing disciplines such as slalom, giant slalom, super-G or downhill and compare the loading of the knee joint and the underlying muscle forces. Further applications of the present simulation framework and musculoskeletal model might be to analyze an out of balance situation during a turning maneuvers. For example, the position of the skier or the ground reaction forces might be perturbed to induced an out of balance situation and analyze the corresponding muscle and ACL forces^36,54^.

In conclusion, the present study presents a computational framework (musculoskeletal model and dynamic optimization) to estimate muscle and ACL forces during turning maneuvers in alpine skiing. ACL forces reached about 0.3 BW on the outside leg during the turning maneuver with the main contribution in the frontal plane due to an external abduction moment. Sagittal plane contributions were low due to consistently high knee flexion (> 60$^{\circ }$), substantial co-activation of the hamstrings and the ground reaction force pushing the anteriorly inclined tibia backwards with respect to the femur. In future research, we will apply the musculoskeletal model to study how the loading of the skier is affected by changes of the equipment of the skier, the skier’s kinematics and neuromuscular control or snow properties. In addition, we are planning to investigate out of balance and injury prone situations and corresponding risk factors.

## Supplementary Information

Supplementary Information 1.

Supplementary Information 2.

## Data Availability

The original contributions presented in the study are included in the article/Supplementary Material. Further inquiries can be directed to the corresponding author.
