# Supplementary material for: Model-based estimation of muscle and ACL forces during turning maneuvers in alpine skiing
Source: Sci Rep. 2023 Jun 3;13:9026. doi: 10.1038/s41598-023-35775-4 (PMC10239490; doi:10.1038/s41598-023-35775-4)
Supplement: Supplementary file 1 — Supplementary Information 1. [file 41598_2023_35775_MOESM1_ESM.pdf]

Supplentary Information

Model-based estimation of muscle and ACL forces during turning maneuvers in alpine skiing

Dieter Heinrich[1,*], **Antonie J. van den Bogert**[2], **Martin Mössner**[1], **and Werner Nachbauer**[1]

[1]University of Innsbruck, Department of Sport Science, Innsbruck, Austria
[2]Cleveland State University, Department of Mechanical Engineering, Cleveland, Ohio, USA
[*]corresponding.author@email.example

S1 Ski-snow contact model

The contact between the skis and the snow was modeled using three force components acting on every ski segment and taking into account the side cut shape of the ski[1]. The three force components were a penetration force acting normal to the snow surface, a shear force acting parallel to the snow surface and orthogonal to the ski edge and finally a friction force.

Specifically, the penetration force F_p was modeled as a function of the penetration depth e and penetration speed $\dot{e}$ of the ski edge orthogonal to the snow surface and the edging angle θ and incorporated a hypoplastic constitutive equation[1]. Following Mössner et al.[1] the penetration force F_p is computed as follows assuming a ski segment is in contact with the snow surface (or setting $e = \max(e, 0)$).

$$F_p(e, \dot{e}, \theta) = HV \cdot f \cdot (1 + \max(D\dot{e}, 0)) \quad (1)$$

with

$$V = \begin{cases} \frac{Le^2}{2tan(|\theta|)} & \text{for} \quad \tan(|\theta|) > \frac{e}{2w} \\ 2Lew & \text{else} \end{cases} \quad (2)$$

and

$$f = \begin{cases} 1 & \text{front part of the ski} \\ \max(\min(\frac{s-s_1}{s_2-s_1}, 1), 0), \ s = \frac{e}{e_{max}} & \text{rear part of the ski} \end{cases} \quad (3)$$

In the equations above, the constants H and D denote the snow hardness and snow damping parameters, respectively; L denotes the length of the ski segment and w the half of the width of the ski segment.

The hypoplastic constitutive equation takes into account that if the penetration depths of the ski segments increase along the ski and the snow is increasingly compressed, the snow remains compressed. A reasonable assumption is, that the maximum penetration depth along the ski occurs at the ski segment centered below the ski binding[2,3]. Therefore, we dived the ski into a loaded front part and an unloaded rear part[2]. In the loaded front part we set f(s) = 1 and in the unloaded rear part the reduction of f(s) due to hypoplasticity is given by equation 3 and the constants s_1 and s_2. In summary, incorporation the hypoplastic constitutive equation into the ski-snow contact model offered the possibility to model the behaviour that during a carved turning maneuver in skiing the front part of the ski typically forms a snow groove and the rear part of the ski follows the groove[1–3].

In the simulation of the turning maneuver, we modified the ski-snow contact forces to be twice differentiable, which is required when using gradient-based optimization. Specifically, we used the following smooth approximations in Table S1, where e_0, D_0, θ_0 and s_0 denote constants. In the smooth approximation of the scaling function f, we introduced the variable $\bar{s}$ to assure that the scaling function f never exceeds 1.

S2 Results

Additional results are shown in Figs. S1 to S5 as well as Table S1 and S2.

original	smooth approximation
$\max(e,0)$	$\frac{1}{2}(e+\sqrt{e^2+e_0^2})$
$\max(D\dot{e},0)$	$\frac{1}{2}(D\dot{e}+\sqrt{(D\dot{e})^2+D_0^2}))$
$\lvert\theta\rvert$	$\sqrt{\theta^2+\theta_0^2})$
$\max(\min(\frac{s-s_1}{s_2-s_1},1),0)$	$\bar{s}^{s_0}, \quad \bar{s}=1-\frac{1}{2}((1-s)+\sqrt{(1-s)^2+s_o^2}$

Table S1. Smooth approximations used in the ski-snow contact model.

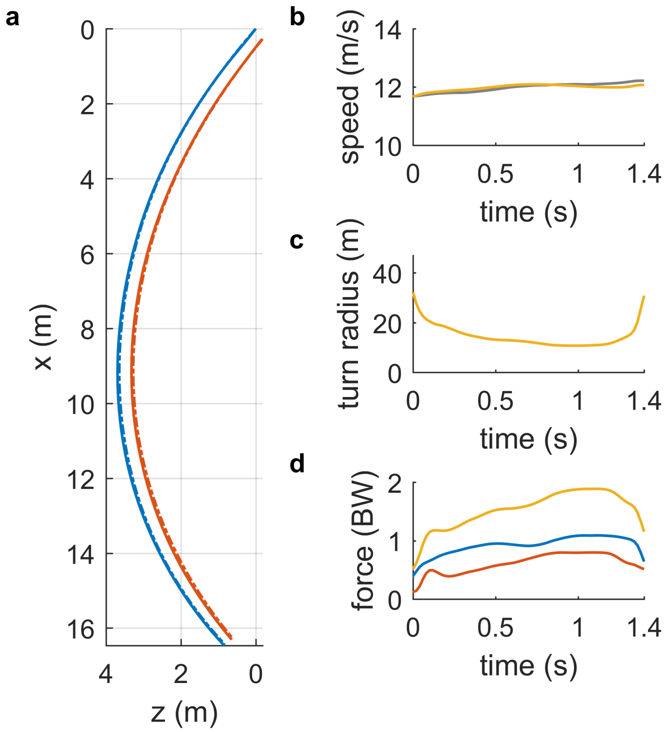

Figure S1. Comparison of the optimized track of the skier in the turning simulation (solid lines) and the corresponding measurement data (dashed lines) in **(a)** as well as the the measured (gray) and optimized speed (yellow) of the skier **(b)** . Additionally, the turn radius of the center of mass of the skier **(c)** as well as the total ground reaction force, the ground reaction force acting on the outside ski (blue) and inside ski (red) are shown **(d)** .

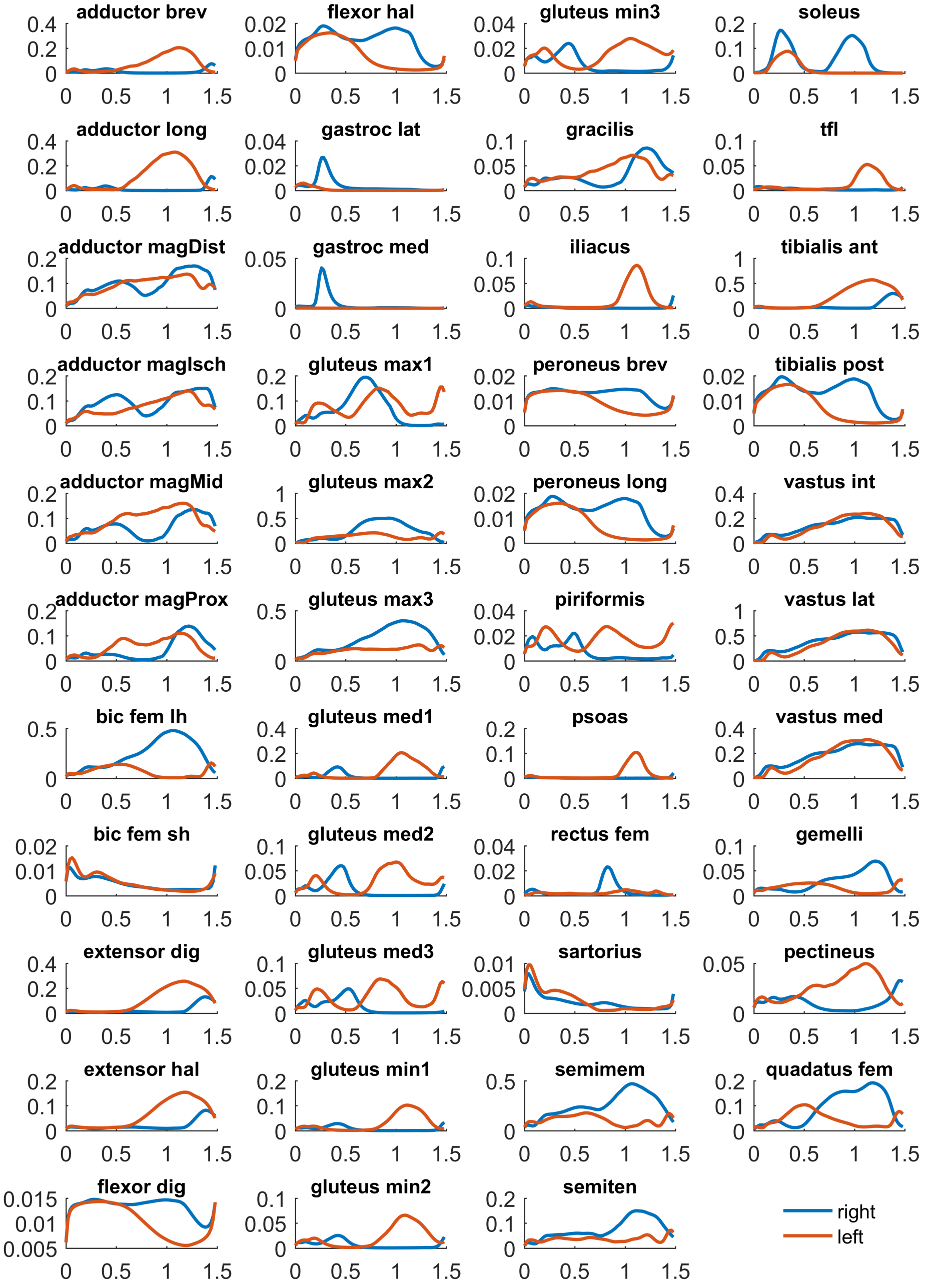

Figure S2. Full set of muscle activation patterns of the muscles at the right outside leg (blue) and left inside leg (red), respectively, during the simulated turning maneuver. Muscle names are taken from the OpenSim models of Cantelli et al.[4] and Harris et al.[5].

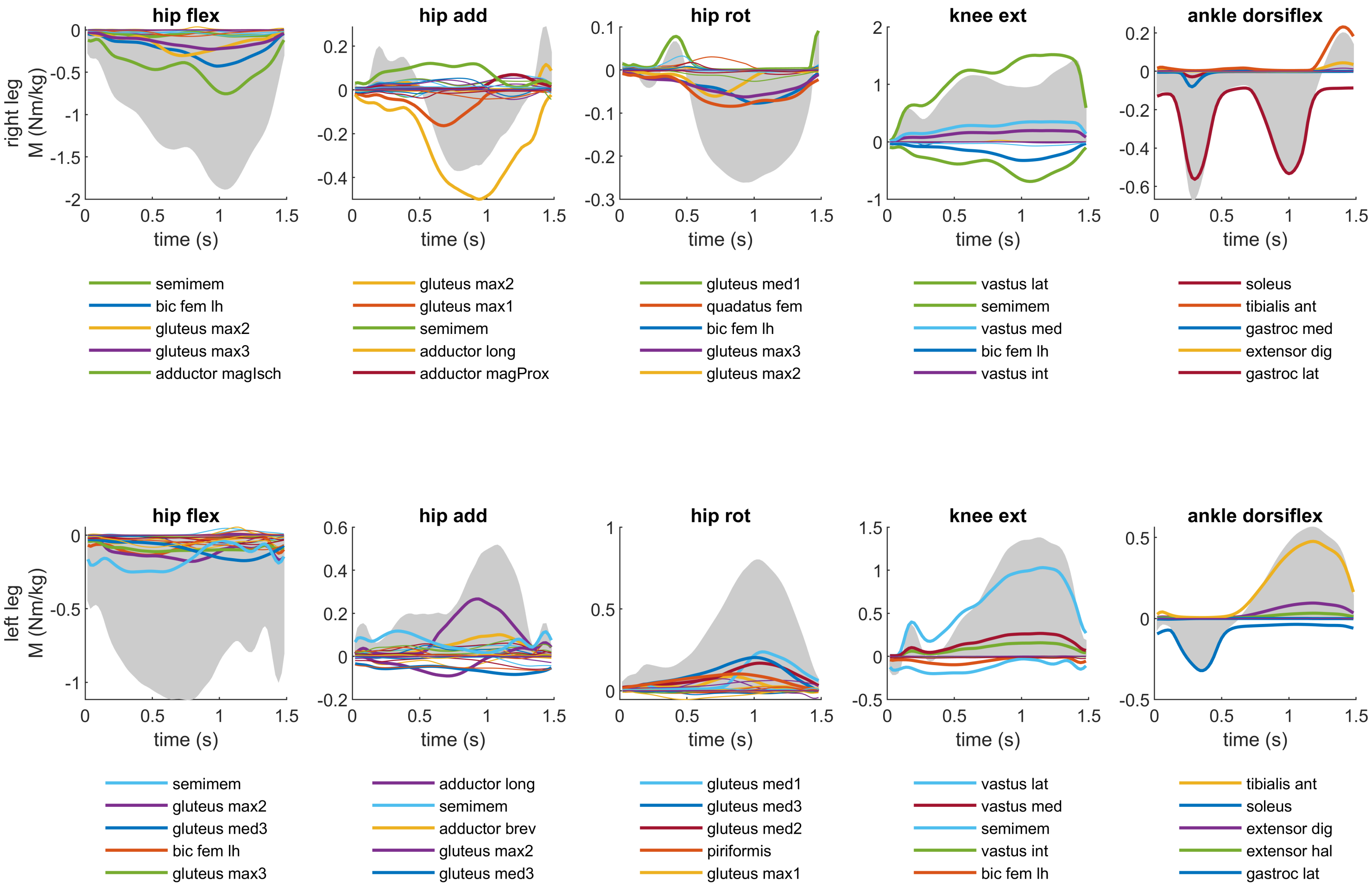

Figure S3. Muscle contributions to the lower-limbs net joint moments (shaded area) during the turning maneuver. The five highest muscle contributions are listed from top to buttom; muscle names are taken from the OpenSim models of Cantelli et al.[4] and Harris et al.[5]. Joint moments were represented as internal joint moments and hip flexion, adduction and internal rotation, knee extension and ankle dorsiflexion moments were denoted as positive.

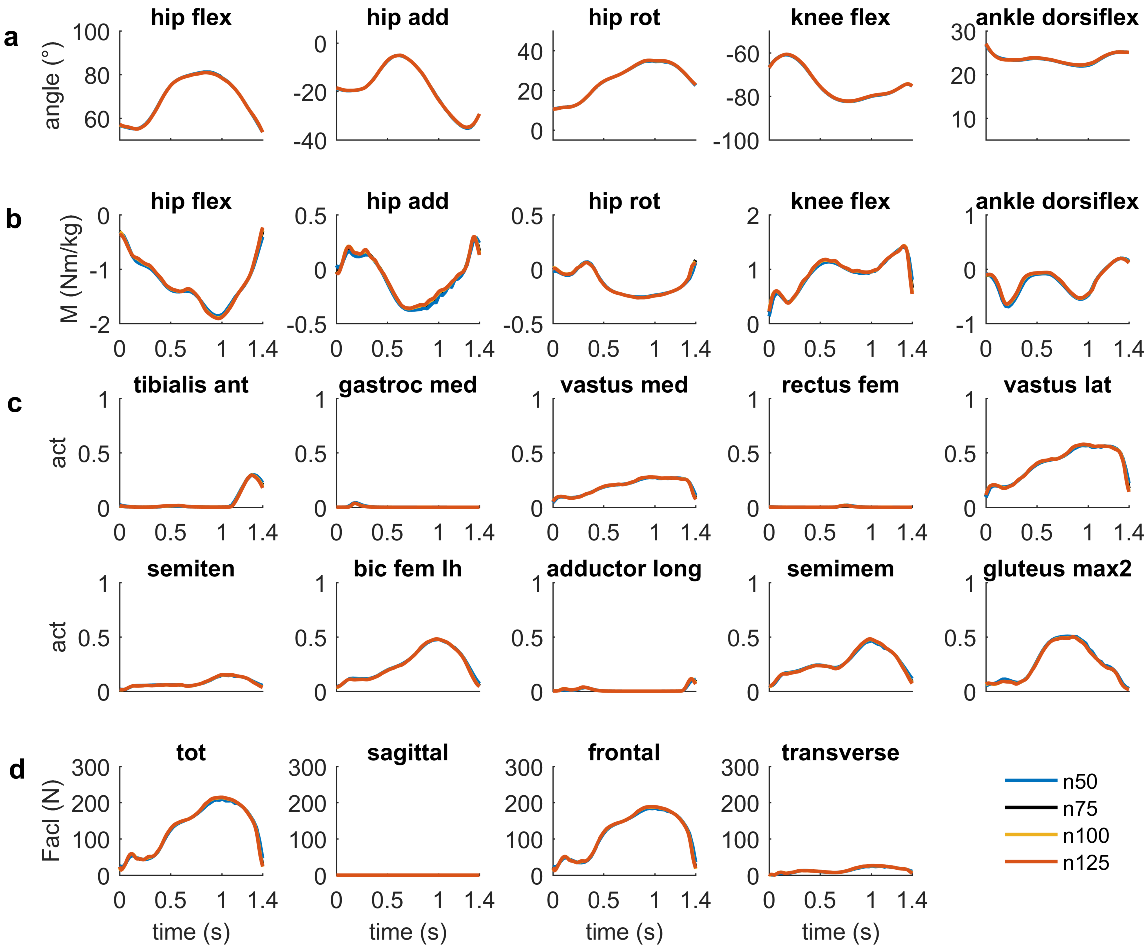

Figure S4. Sensitivity study analyzing the effect on the joint angles (a), joint moments (b), muscle activation patterns (c) and ACL forces (d) of the right outer leg using 50 (n50), 75 (n75), 100 (n100) and 125 (n125) mesh points in the optimization.

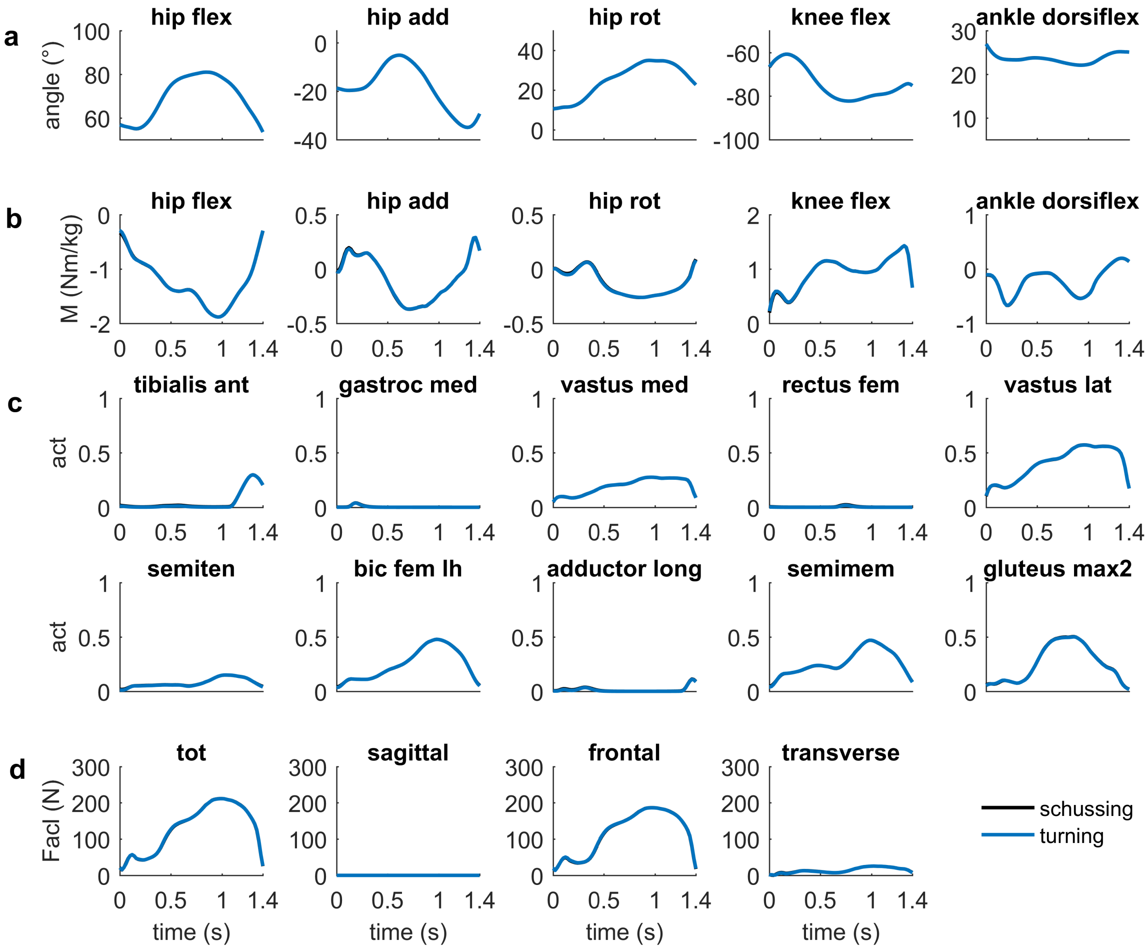

Figure S5. Sensitivity study analyzing the effect of the choice of the initial guess in the optimization on the joint angles (a), joint moments (b), muscle activation patterns (c) and ACL forces (d) of the right outer leg. The initial guesses were a data-independent schussing simulation (schussing) as well as turning simulation (turning), that was computed using a PD controller to track the experimental joint angles of the skier followed by a muscle redundancy solver.

sensitivity study	objective function J tracking error (w_1)	muscle effort (w_2)	regularization (w_3)
a^2	0,085	0,219	0,002
a^3	0,076	0,121	0,002
a^5	0,066	0,061	0,002
w2.5	0,052	0,076	0,002
w5	0,065	0,129	0,002
w10	0,085	0,219	0,002
w20	0,120	0,372	0,002
w40	0,183	0,621	0,003
schussing	0,085	0,219	0,002
turning	0,085	0,218	0,002
n50	0,097	0,219	0,004
n75	0,085	0,219	0,002
n100	0,080	0,218	0,001
n125	0,078	0,218	0,001

Table S2. Results of the sensitivity study regarding the three terms (i.e., tracking error, muscle effort, regularization) in the objective function J. The nominal values of the weighting coefficient were $w_1 = 1$, $w_2 = 10$, $w_3 = 1$. In the sensitivity study, the number of nodes (n50, ..., n125), the initial guess (schussing, turning), the weighting coefficient (w2.5, ..., w40) of the muscle effort term (i.e., w_2) and the exponent of a in the muscle effort term (a^2, a^3, a^5) were varied.

References

1. Mössner, M. *et al.* Modeling the ski-snow contact in skiing turns using a hypoplastic vs an elastic force-penetration relation: Modeling the ski-snow contact in skiing turns. *Scand. J. Medicine & Sci. Sports* **24**, 577–585, DOI: 10.1111/sms.12035 (2014).

2. Federolf, P., Roos, M., Lüthi, A. & Dual, J. Finite element simulation of the ski–snow interaction of an alpine ski in a carved turn. *Sports Eng.* **12**, 123–133, DOI: 10.1007/s12283-010-0038-z (2010).

3. Heinrich, D., Mössner, M., Kaps, P. & Nachbauer, W. Calculation of the contact pressure between ski and snow during a carved turn in Alpine skiing: Calculation of contact pressure. *Scand. J. Medicine & Sci. Sports* **20**, 485–492, DOI: 10.1111/j.1600-0838.2009.00956.x (2009).

4. Catelli, D. S., Wesseling, M., Jonkers, I. & Lamontagne, M. A musculoskeletal model customized for squatting task. *Comput. Methods Biomech. Biomed. Eng.* **22**, 21–24, DOI: 10.1080/10255842.2018.1523396 (2019). PMID: 30398067, https://doi.org/10.1080/10255842.2018.1523396.

5. Harris, M. D. *et al.* Higher medially-directed joint reaction forces are a characteristic of dysplastic hips: A comparative study using subject-specific musculoskeletal models. *J. Biomech.* **54**, 80–87, DOI: 10.1016/j.jbiomech.2017.01.040 (2017).
